# Plasma-based one-step synthesis of tungsten oxide nanoparticles in short time

**DOI:** 10.1038/s41598-023-34612-y

**Published:** 2023-05-08

**Authors:** F. Baharlounezhad, M. A. Mohammadi, M. S. Zakerhamidi

**Affiliations:** Faculty of Physics, University of Tabriz, Tabriz, Iran

**Keywords:** Plasma physics, Nanoscience and technology, Physics

## Abstract

Nanostructured tungsten oxide as a semiconductor metal oxide has attracted considerable attention due to its promising and notable properties. Tungsten oxide nanoparticles can be used in a wide range of technology and applications such as catalysts, sensors, supercapacitors, etc. In this study, nanoparticles were prepared via a simple method using an atmospheric glow discharge. This modern approach had many advantages such as high efficiency and straightforward function. Synthesis performance was done in only one step and a short time which started at 2 min and had been continued for 8 min. The X-ray diffraction pattern revealed the formation ${\mathrm{WO}}_{3}$ at atmospheric pressure. The synthesized particle size was characterized using scanning electron microscopy. According to the experimental results, the synthesis was greatly influenced by the applied voltage, gas type, and plasma forming side over the water surface. Increases in electrical potential difference and thermal conductivity of the gas led to a greater rate of synthesis, while this rate was reduced by decreasing the atomic weight of the gas.

## Introduction

Nanoparticles have been extensively used due to their unique optical properties, shape, and size. Biological, chemical, and physical methods are common synthesis methods for these particles^1,2^. Metal nanoparticles (MNPs) are attracting the attention of scientists for their adjustable properties for use in a wide range of applications, including biomedicine, the electronic industry, and optical devices^3–6^. A crystalline powder made of metal nanoparticles, such as tungsten oxide, also known as tungsten trioxide (${\mathrm{WO}}_{3}$), can be utilized in electrochemistry, photocatalysts, smart windows, and electronic devices^7–10^.

Nanotechnology-related research and development have accelerated globally. One of their key products is metallic nanoparticles (MNPs). Nanoparticles are most commonly synthesized by wet chemical techniques. These create nucleation with the assistance of reducing chemical agents in the solution^11^. In comparison, synthesis through plasma prepares the nucleus without chemical agents or overlying agents. In non-thermal plasmas (NTP) ions and electrons, are at different temperatures^12^. In this regard, the non-thermal synthesis of nanoparticles can be enabled at various melting temperatures. According to the Hall-Patch relationship, a strength can be obtained like material theoretical strength by reducing the grain size. NTP technology, as a prominent clean and easy synthesis method of nanomaterials, has attracted a lot of attention due to its specific properties in reducing grain size^13^.

Ashkarran et al.^14^ synthesized ${\mathrm{WO}}_{3}$ nanoparticles by the arc electric discharge method in deionized water with different arc currents and investigated the properties of the resulting nanoparticles. The particle size in the 25 A arc current was about 30 nm. The particle size increased with increasing arc current to 64 nm, which caused a decrease in the band gap from 2.9 to 2.6 eV. Prepared samples at the lowest current had more photocatalytic activity because of the smallest particle size and highest surface area. Chen et al.^15^ prepared ${\mathrm{WO}}_{3}{.\mathrm{H}}_{2}\mathrm{O}$ nanoparticles with a size of about 5 nm by pulsed plasma in deionized water. The quenching effect and liquid environment inherent in pulsed plasma inside deionized water yielded ultra-small particles with lattice lengths larger than those of reference lattices. The ${\mathrm{WO}}_{3}{.\mathrm{H}}_{2}\mathrm{O}$ showed higher absorption than ST-01 ${\mathrm{TiO}}_{2}$ and Wako ${\mathrm{WO}}_{3}$ nanoparticles in the visible region. Sirotkin et al.^16^ used an underwater shock discharge to synthesize ${\mathrm{WO}}_{3}$ nanoparticles, which formed a monoclinic modification ${\mathrm{WO}}_{3}$ with an average particle diameter of about 60 nm, depending on the discharge current and additional electrolytes. The sample displayed high photocatalytic activity due to the low band gap and porous structure. Ranjan et al.^17^ synthesized ${\mathrm{WO}}_{3}$ nanoparticles by a wire explosion process in an oxygen environment and investigated their photocatalytic behavior. The particle size followed a log-normal distribution with a minimum mean size of 24.1 nm. The band gap of nanoparticles was measured to be 2.92 eV. Chang et al.^18^ produced nano-tungsten colloids $({\mathrm{W}}_{2.00}\mathrm{ and W})$ with an average particle size of 164.9 nm, absorbance wavelength of 315 nm, $\upzeta$ potential of − 64.9 mV, and a minimum particle size of 11 nm using a spark discharge system in deionized water.

In this study, the synthesis of tungsten oxide nanoparticles has been reported using NTP resulting from an atmospheric pressure glow discharge that interacted with the water surface. This method provides a one-step and rapid procedure for the synthesis of massive amounts of ${\mathrm{WO}}_{3}$ nanoparticles with high efficiency in a relatively short time. This technique of creating ${\mathrm{WO}}_{3}$ nanoparticles is unique in that it produces NP outside of water solvent media, doing away with the necessity to remove the nanopowder from the solution following the creation process. The synthesized product was analyzed by XRD and SEM.

## Experimental setup

**Figure 1:**
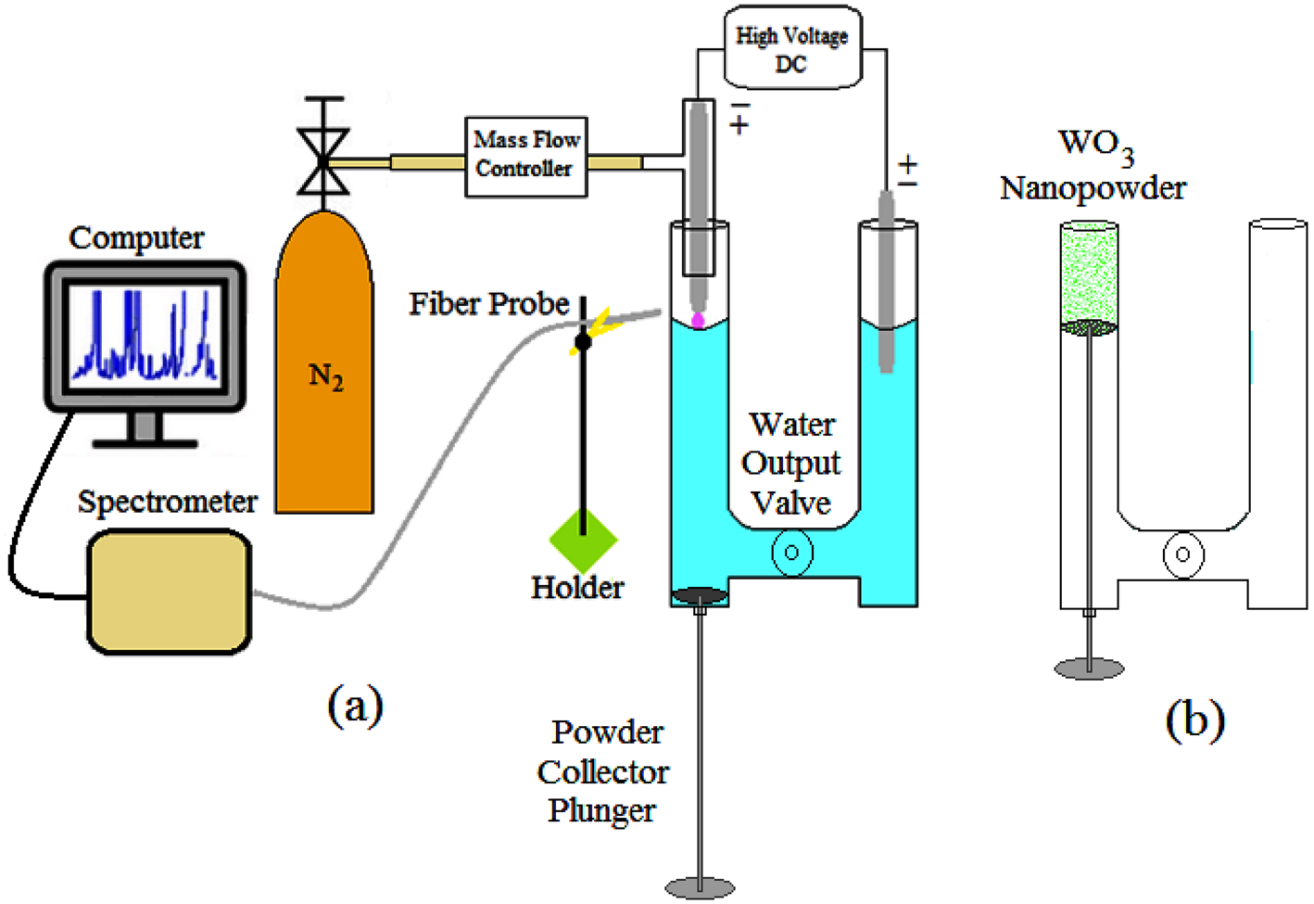
Experimental setup in the synthesis of nanoparticles.

The experimental setup during plasma production and the synthesis of nanoparticles and the method of collecting the synthesized nanoparticles are shown in Fig. 1a,b respectively. In the present study, a Hoffman electrolysis reactor including a collecting piston on the cathode side was used for nanoparticle synthesis. The anode and cathode were tungsten rods with a diameter of 1.5 (mm). The reactor was filled with water (pH = 6.68, EC = 101). The power supply was DC at 7 (kV). Plasma was produced by nitrogen gas and the mass flow controller was adjusted to 50sccm. The nanopowder was collected by moving the plunger upwards after discharge and emptying the reactor water through the output valve. Spectroscopy was done by TIDA spectrometer (UCS-G400) in the wavelength range (200–1000 nm) made by Teksan Company. Morphology and elemental analysis were performed through field emission scanning electron microscopy (MIRA3 FEG-SEM), and the crystal structure of the sample was done via X-ray diffraction (TD-3700).

## Result and discussion

### The plasma-water interaction

Although there are different configurations that can be used for plasma interaction with liquid, the main focus of this work was on water because of its high heat capacity, which served as an electrode in the DC plasma circuit and kept the plasma almost cold. A DC, negatively-biased plasma interacted with the water anode electrode. By radiating electrons from the cold atmospheric plasma toward the water's surface on the cathode side, a significant number of electrons were exposed above the water's surface. These electrons were stabilized as hydrated electrons. In the cathodic plasma configuration, hydrated electrons also made it to the water surface and were solvated, which may be significant in a number of processes. Water molecules were energized, broken down, or ionized as a result of the reaction between these high-energy electrons and the molecules. The second-order recombination of ${\mathrm{e}}^{-}$, which corresponds to hydrogen evolution in the electrolytic system, was created when the hydrated electrons that were dissolved in the water with low energy reacted with the water molecules^19,20^.

Of course, it should be noted that the primary ionic species of N, O, and Ar are made in the gas phase plasma, which can undergo several conversions. Secondary species result from the degradation or interaction of the primary species with each other or molecules. These conversions are created in the interface layer where plasma discharges pass through to the water surface. However, in the case process of our investigation, only the presence of primary species was ineffective, and switching from the cathode to the anode of the plasma-producing electrode failed to produce any tungsten oxide nanoparticles.

### The mechanism of plasma generation and plasma-based synthesis of tungsten oxide

There are different types of plasma-liquid systems, which are categorized as having plasma either over or inside the liquid. In both types, there is a plasma-liquid interface. The interface region is a very important zone for synthesizing nanoparticles because it is where the main physical and chemical reactions can happen. This study used an atmospheric plasma system over the water for tungsten oxide nanoparticle preparation. The anode electrode was placed above the water surface, and the cathode electrode was placed inside the water. Plasma is known as anodic plasma in this design^21^. Then, the process was reversed, which is called cathodic plasma^21^. In this case, the non-contact electrode completed the circuit without making contact with the water surface.

The gas type and plasma formation sides strongly affect the synthesis process. In nitrogen cathodic plasma, a significant amount of nanoparticles were synthesized at 7 kV in a very short time, 8 min. In similar circumstances, synthesized nanoparticles were fewer in oxygen cathodic plasma compared to nitrogen cathodic plasma. Anodic plasma did not lead to the synthesis of tungsten oxide nanoparticles in nitrogen and oxygen. Tungsten oxide nanoparticles were not produced by argon in both cathodic and anodic plasma at 8 min. The inert gas reduced the evaporation of the tungsten electrode and prevented its oxidation. On the other hand, argon has low thermal conduction and a high atomic weight. High atomic weight reduces thermal conduction and convection. The presence of oxygen or nitrogen gas, due to their high thermal conduction and low atomic weight, led to the conduction of heat to the electrode surface, which evaporated tungsten and caused its oxidation. However, as the voltage and time increased, synthesis was also done in argon cathodic plasma. According to these interpretations, nitrogen cathodic plasma was chosen for the synthesis and analysis of tungsten oxide nanoparticles.

Applying the potential across the electrodes caused the gas breakdown and formed stable glow discharge plasma between the cathode and the water surface (interface) at atmospheric pressure. In other words, plasma was substituted with the metal cathode electrode in the system at a direct current bias. Most of the electrons from the cathode entered the interface. Energetic electrons were able to excite and ionize particles (molecules and atoms) in this region. Due to the ionization in the plasma-liquid interface, many secondary electrons were produced and positive ions. When the water acted as the anode, the voltage at the interface did not reduce, and the secondary electrons fell from the plasma bulk to the water. The secondary electrons were dissolved in water and formed hydrated electrons $({\mathrm{e}}_{\mathrm{aq}}^{-})$. The resulting nitrogen-positive ions moved toward the cathode. The negative bias surface of the cathode was bombarded with nitrogen-positive ions resulting from electron collision, and kinetic energy was transferred to its surface. This caused the momentum transferring and erosion of the tungsten cathode. In other words, the collision of energetic ions with the cathode surface led to sputtering. After the collision of positive ions, it could produce other electrons to maintain the discharge.

**Figure 2:**
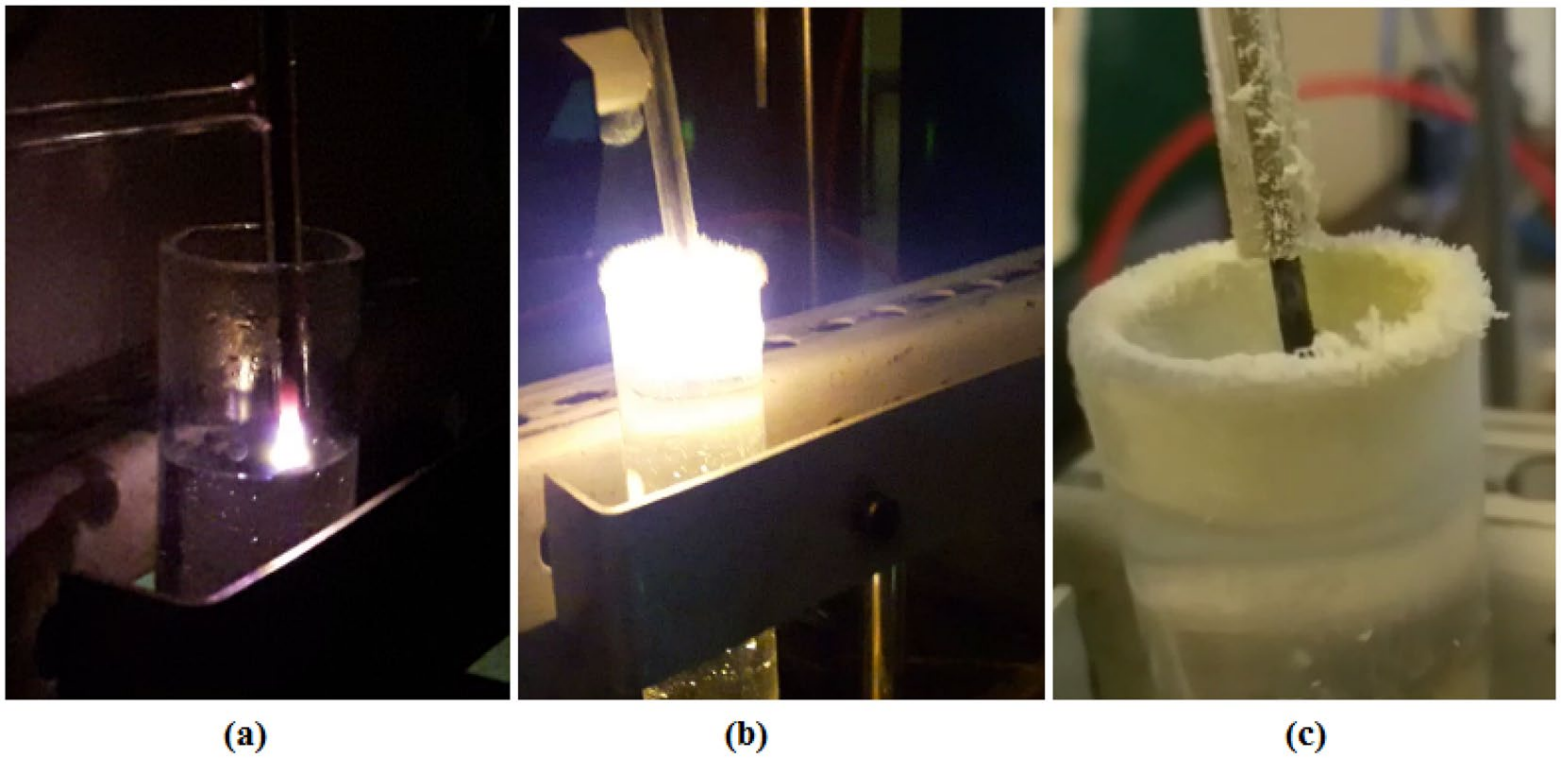
(**a**) Start (**b**) after 3 min, and (**c**) end of atmospheric sputtering to produce tungsten oxide nanopowder.

According to Fig. 2, in the non-contact cathode model, ${\mathrm{WO}}_{3}$ nanoparticles started to form over the hollow part of the glass wall of the Hoffman reactor, the cathode, and the water surface after 2 min.

Depending on the impact angle, the energy, the mass of the collisional ions, and the bond energy of the cathode atoms, the incident ions can be reflected, adsorbed on the surface, implanted the atoms on the cathode surface, or removed. Incident ions can excite cathode atoms to emit secondary electrons. They subsequently got away from the cathode, ionized the neutral gas atoms, and assisted in maintaining the plasma. In this process, the cathode tungsten material was released as atomic vapor. Tungsten atoms accelerated in the plasma-generating field. They reacted with negative ions of oxygen dispersed from the metal oxide cathode with different orientations, and then covered the glass wall reactor in a thin layer of ${\mathrm{WO}}_{3}$ powder.

Furthermore, the formation of plasma drove oxidation–reduction reactions by highly reactive species introduced into the water. By contacting plasma with the water surface, energetic plasma species such as free electrons, ions, or electronically excited atoms dissociated molecules of either the water surface or vapor, which provided the necessary oxygen for tungsten oxidation.

Sputtering and evaporation are two physical processes that occurred for the synthesis of nanoparticles in this plasma interaction system with the water surface. In this case, the synthesis efficiency was 78%. For 1.285 gr of erosion of the cathode, 1 gr of tungsten oxide was synthesized on the surface of the reactor glass wall in 8 min.

### Spectral investigation of anodic and cathodic plasmas

**Figure 3:**
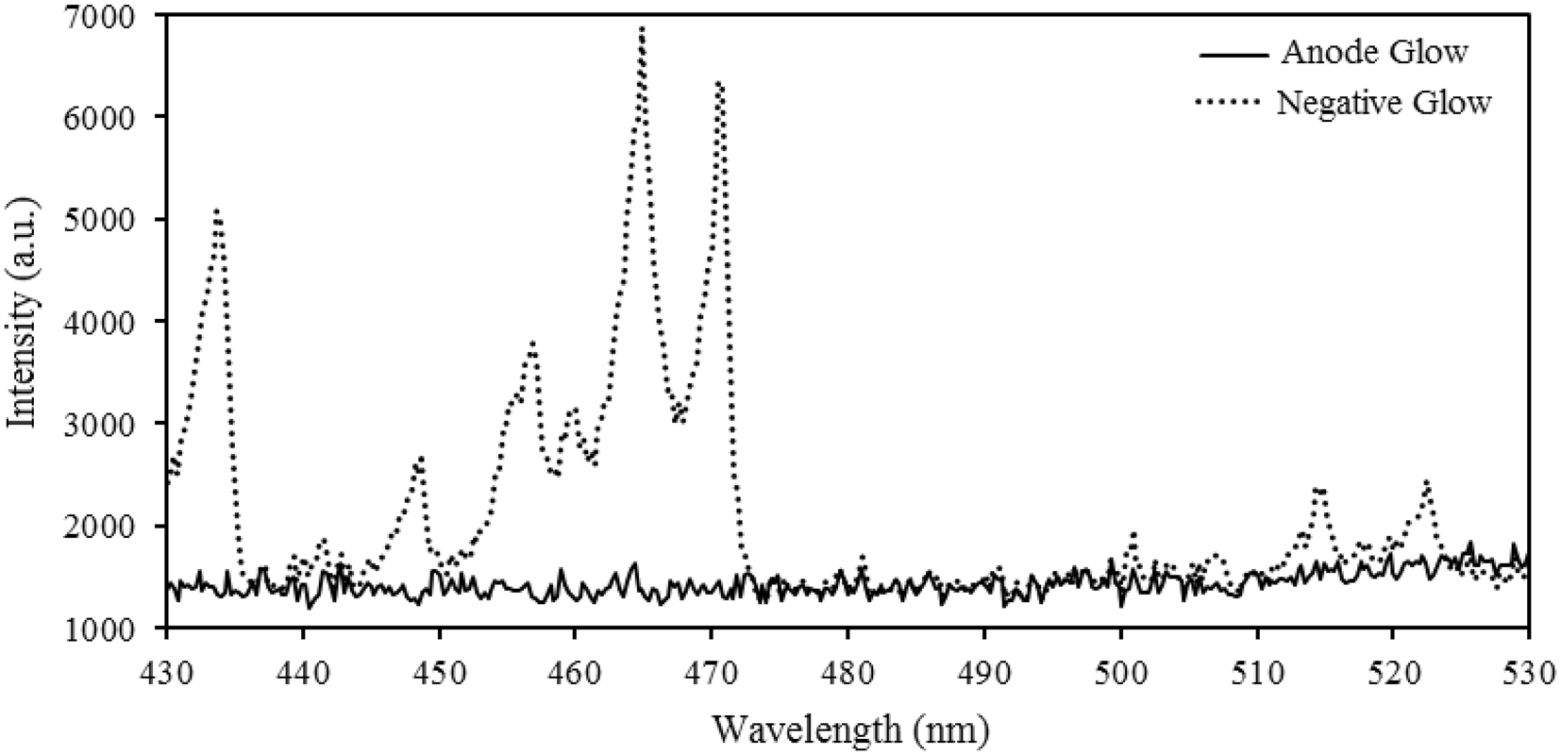
Spectra of plasma emission lights in anode glow and negative glow.

If an incident electron gives enough energy to a nitrogen atom, it ejects one of the electrons attached to it. So it results in an ionized nitrogen atom. The excitation of an orbital electron to a higher state decays to the ground state with the emission of photons. Many electron-atom collisions are yielded. As a result, considerable light emits between the cathode and anode electrodes in the gas discharge. The light emission has the highest intensity in the negative glow at the cathode side and the anode glow at the anode side^21^. Therefore, the two mentioned regions with strong emissions around the anode (at anodic plasma) and the cathode (at cathodic plasma) were selected for spectroscopy. These regions had been separated from the electrodes by weak emission or no emission zones. Figure 3 shows the spectra of plasma emission lights in the anode glow and the negative glow regions in anodic and cathodic plasmas, respectively.

**Figure 4:**
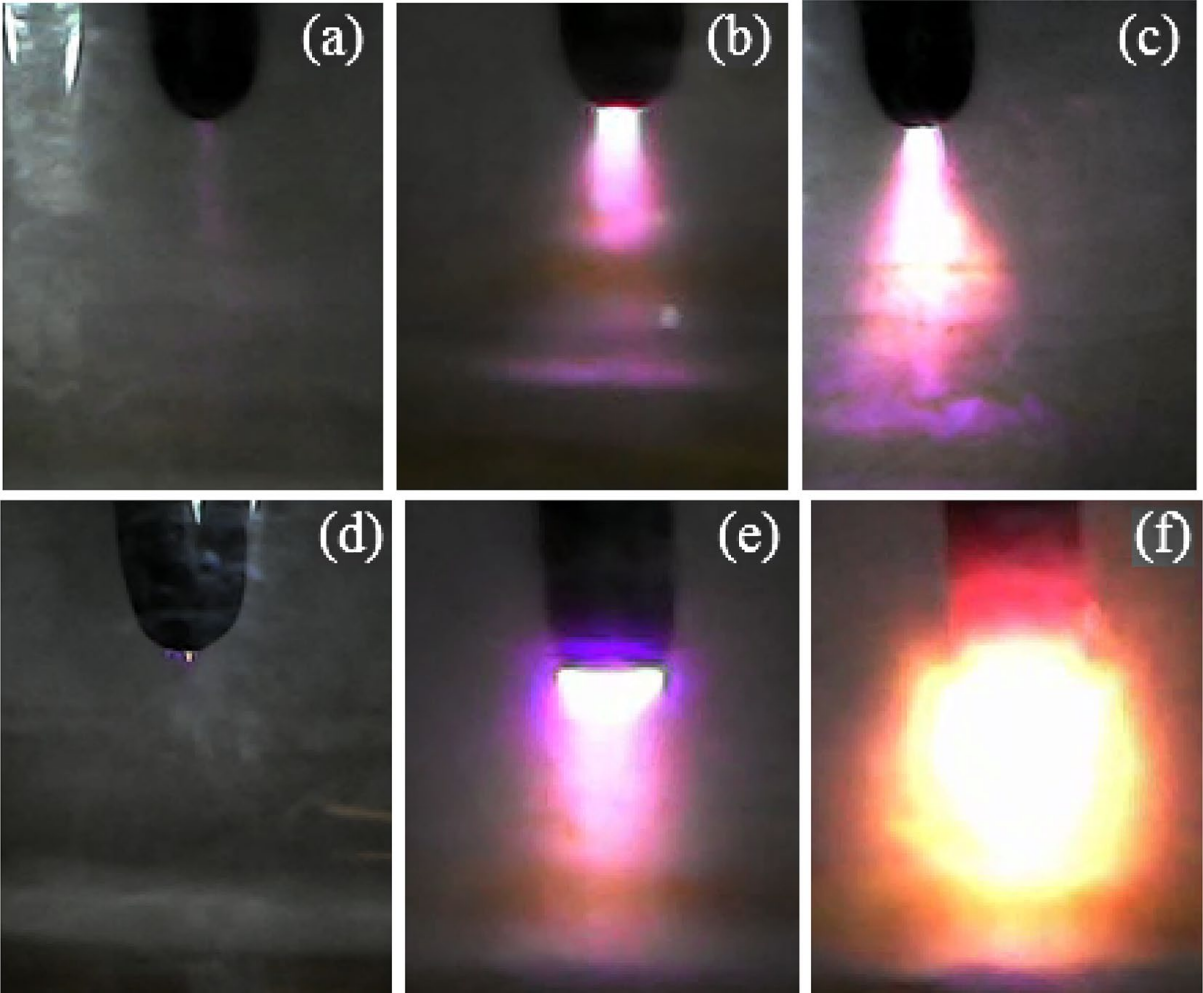
Anodic plasma at voltages at (**a**) 3 kV, (**b**) 4 kV, (**c**) 7 kV and cathodic plasma at voltages at (**d**) 3 kV, (**e**) 4 kV, (**f**) 7 kV on the water surface.

The emission intensities in the negative glow were much higher than in the anode glow. In the anode glow, electrons with lower energy form a bright region with less intensity than the negative glow due to collisions with liquid atoms and molecules. In the negative glow, electrons with high density and low speed easily recombine with positive ions and emit high-intensity recombination radiation. In other words, electron energy in atmospheric glow discharge with a liquid electrode was obtained more in the negative glow than in the anode glow, as shown in Fig. 4.

### Characterization of tungsten oxide

**Figure 5:**
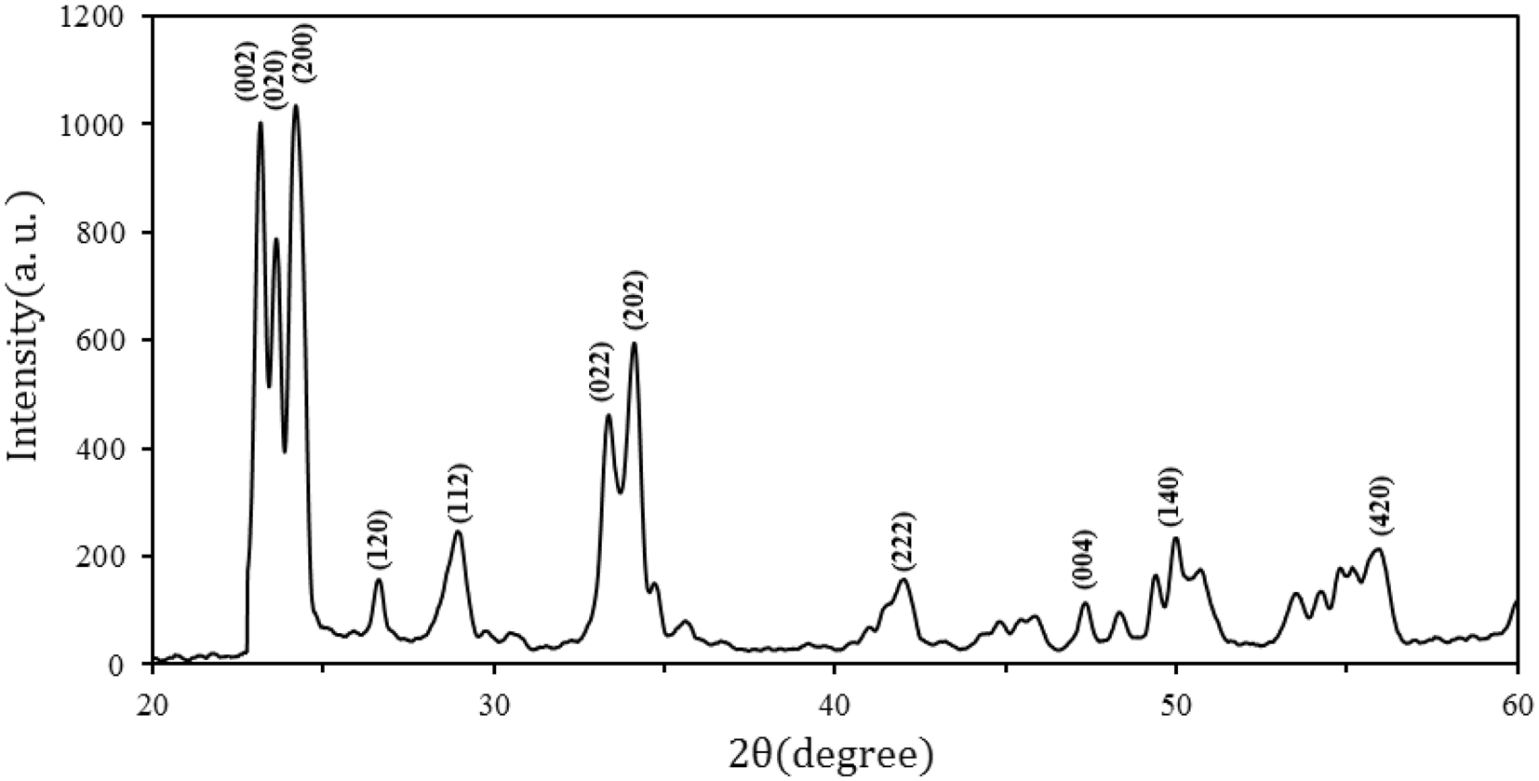
X-ray diffraction spectrum of ${\mathrm{WO}}_{3}$ nanoparticles.

The X-ray diffraction pattern of synthesized nanoparticles was presented in Fig. 5. Sharp peaks were identified at $2\uptheta =23.20$, $2\uptheta =23.67$, $2\uptheta =24.43$, $2\uptheta =33.37$, and $2\uptheta =34.22$ degrees corresponding monoclinic ${\mathrm{WO}}_{3}$ phase (space group: P21/n(14)).

The crystal domain size of ${\mathrm{WO}}_{3}$ for the broadening of the prominent peak (200) was estimated using the famous Debye–Scherer’s Eq. (1)^22^

1 $$\mathrm{D}=\frac{\mathrm{k\lambda }}{\mathrm{\beta cos\theta }}$$

Here D is the volume average crystallite size of the particles, λ is the X-ray wavelength, β is (FWHM) of the diffraction peak, θ is the diffraction angle and k is Debye–Scherer’s constant. D was obtained by using the width of the largest and most symmetrical peak equal to 19.40(nm). The value of the dislocation density, ${\updelta }_{\mathrm{np}}$, shows the degree of crystallinity of the nanoparticle profile and was calculated by Eq. (2)^23^,

2 $${\updelta }_{\mathrm{np}}=\frac{1}{{\mathrm{D}}^{2}}$$

Dislocation density obtained 0.003 ${\mathrm{nm}}^{-2}$.

**Figure 6:**
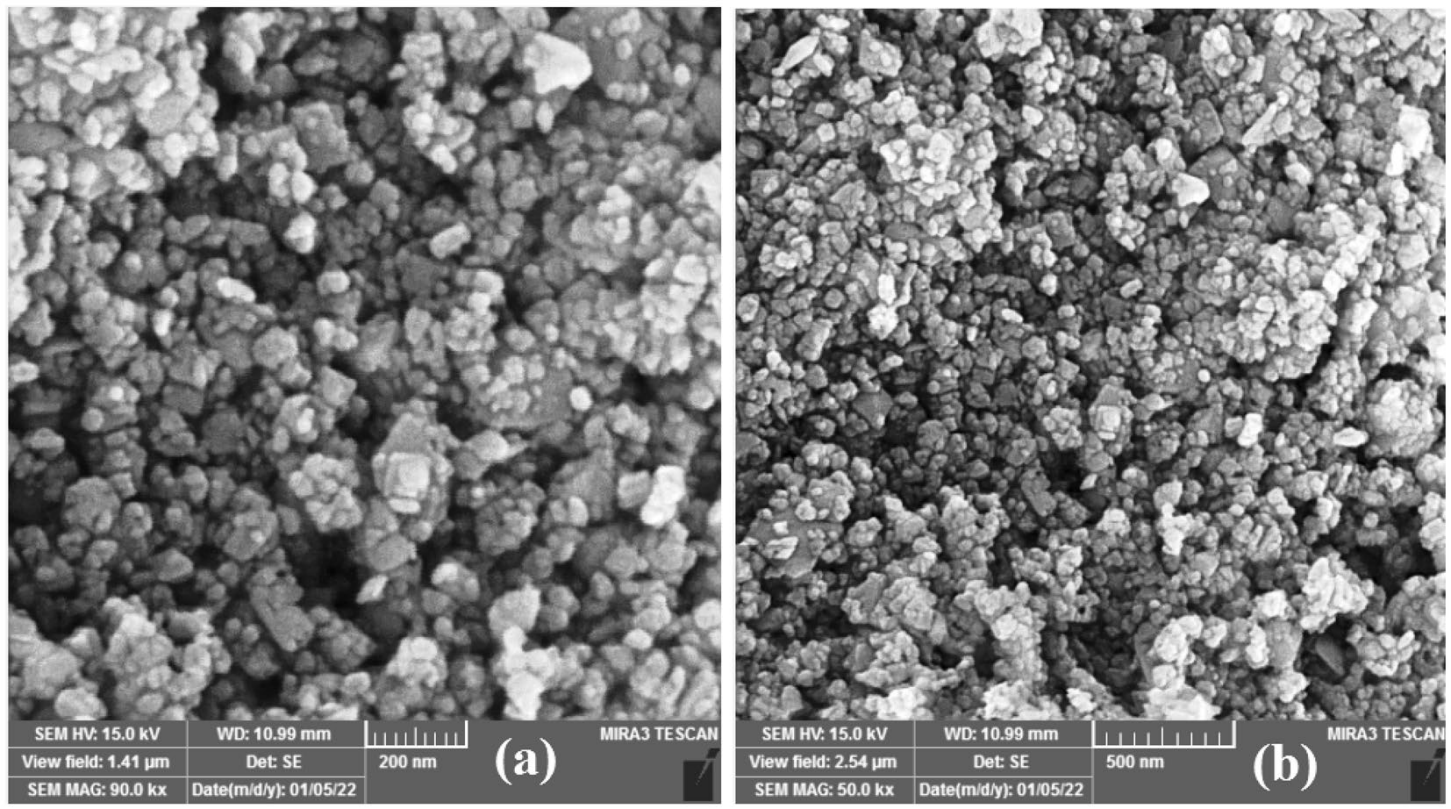
(**a**) and (**b**) SEM images of synthesized tungsten oxide nanopowder at room temperature and atmospheric pressure.

**Figure 7:**
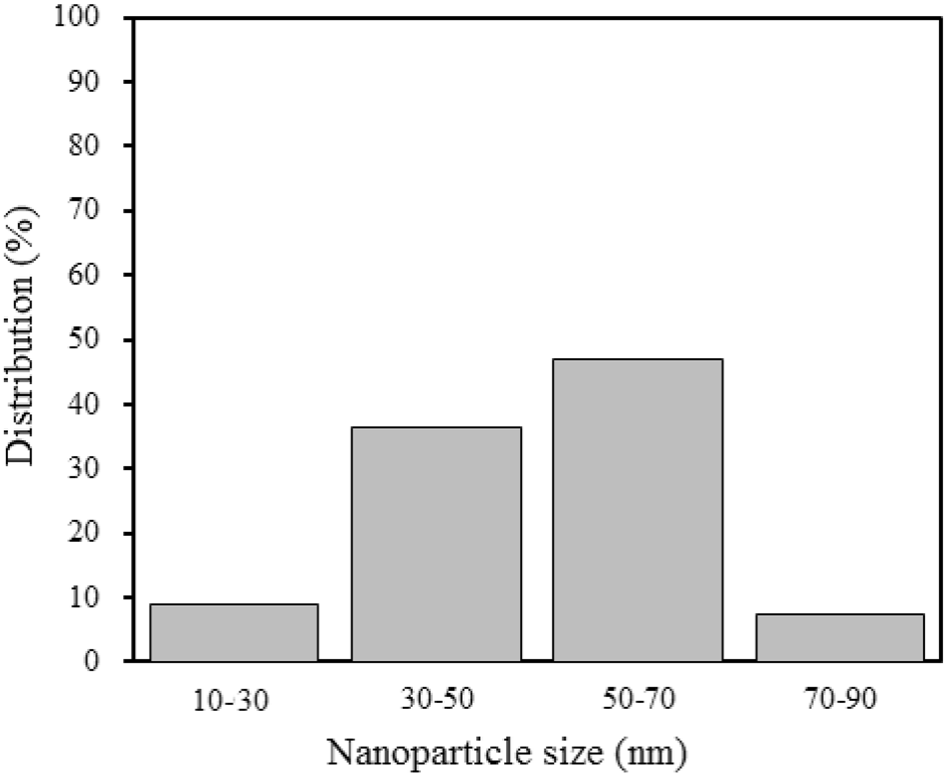
Particle size distribution histogram determined from the SEM images.

To study the morphology of nanoparticles, images of the SEM technique shown in Fig. 6 were used. Figure 6 shows that the nanoparticles are well separated and quite small in size, although large chunks of nanoparticles are occasionally observed. Particle size distribution correlated to the SEM image is displayed in Fig. 7. The average diameter of the ${\mathrm{WO}}_{3}$ MNPs was estimated to be 50–70 (nm). The total number of nanoparticles was 200. Discrepancy observed between crystallite size and SEM showed synthesized particles were not perfectly single crystallite and had several dislocations, which disconnected the periodicity of the crystalline nature. As a result, a particle contained some crystallites as coherently diffracting regions. So each particles was agglomeration several crystallites stuck together, which caused the particle size to be larger in the SEM image analysis.

## Conclusion

Although plasma-liquid setups have formerly been used very well for nanomaterials processing and synthesis in addition to surface functionalization, more efforts should be made to optimize and expand the role of plasma-liquid interactions in this field with a comprehensive understanding of plasma-induced physical and chemical properties. Gathering this knowledge can lead to new potential use for plasma-liquid interactions in a variety of fields, including nanoparticles. In this study, an atmospheric glow discharge technique was used to synthesize tungsten oxide nanopowder. In the future, it is anticipated that adjusting experimental parameters will offer a method for modifying the size of the nanoparticles. Eventually, the following conclusions were drawn from this study:

i. The ${\mathrm{WO}}_{3}$ nanoparticles were obtained with a radius in the range of 50–70 nm from SEM results.
ii. This technique offers an easy, low-cost, and environmentally friendly way to fabricate large quantities of tungsten trioxide nanoparticles in a very short time.
iii. The gas and plasma types and the applied voltage play a significant role in the synthesis of nanoparticles. With increasing voltage, the probability of synthesis in cathodic plasma increases. Nitrogen cathodic plasma has the highest synthesis efficiency in comparison to oxygen and argon cathodic plasmas.
iv. The amount of synthesis in cathodic plasma increases in the presence of gases with high thermal conductivity and low atomic weight.

## Supplementary Information

Supplementary Information.

## Data Availability

The data sets generated during and/or analyzed during the current study are available in the OSF repository, [https://osf.io/mfqw6/?view_only=8c4ab06a6b3d4731960199eb23e4749d].
