# Supplementary material for: Plasma-based one-step synthesis of tungsten oxide nanoparticles in short time
Source: Sci Rep. 2023 May 8;13:7427. doi: 10.1038/s41598-023-34612-y (PMC10167255; doi:10.1038/s41598-023-34612-y)
Supplement: Supplementary file 1 — Supplementary Information. [file 41598_2023_34612_MOESM1_ESM.pdf]

Supporting Information

Plasma-based one-step synthesis of tungsten oxide nanoparticles in short time

F.Baharlounezhad[1], M.A. Mohammadi[1*], M.S. Zakerhamidi[1]

1. Faculty of Physics, University of Tabriz, Tabriz, Iran

** Corresponding author:*

Mohammad Ali Mohammadi

Tel: number: +98-41-33393323

Fax: number: +98-41-33341244,

E-mail: mohammadidorbash@yahoo.com and m_a_mohammadi@tabrizu.ac.ir

Electron temperature in nitrogen plasma was determined by examining the emission intensities in the wavelength range of 430-530nm around the anode and cathode. It was calculated by the Boltzmann plot method according to equation (1) equal to 0.568eV and 0.636eV for anode and cathode respectively [1]

$$\ln \frac{I_{ji}\lambda_{ji}}{A_{ji}g_j} = -\frac{E_j}{k_B T} + c \qquad (S1)$$

In Fig. S1, the Boltzmann plot of N I spectral lines is shown.

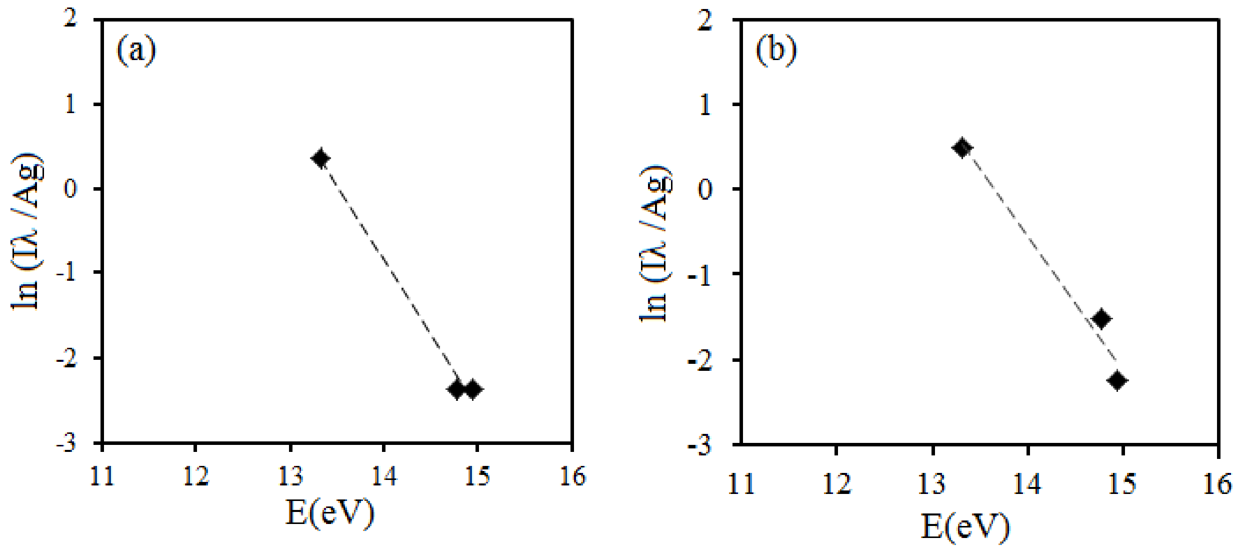

Fig S1. The Boltzmann plot of N I spectral lines of (a) anodic plasma and (b) cathodic plasma.

Table S1 demonstrates the atomic data of N I spectral lines [2].

Table. 1: Atomic data of chosen N I lines.

Wavelength (nm)	Statistical Weight	Transition Probability $(10^8 s^{-1})$	Upper Level Energy (eV)
439.240	4	0.0176	14.948
467.068	4	0.0180	14.780
518.140.	4	0.0144	13.322

Electron density was measured by analyzing the full width at half maximum (FWHM) in Stark broadening of the hydrogen Balmer H_β spectral line. The FWHM of the Stark broadening is determined by equation (2) [1]

$$\Delta\lambda_{\mathrm{stark}} = 4.8(\mathrm{nm})\left(\frac{\mathrm{n_e}}{10^{23}}\right)^{0.68116} \tag{S2}$$

This broadening is related to the Lorentz and the Van der Waals broadening via equation (3) [20]

$$\Delta\lambda_{\mathrm{stark}} = \Delta\lambda_{\mathrm{Lorentz}} - \Delta\lambda_{\mathrm{Vander\ Waals}} \tag{S3}$$

The Lorentz broadening can be estimated by the fitting of experimental spectra with the Voigt profile. FWHM of the Vander Waals broadening is determined as follows [1]

$$\Delta\lambda_{\mathrm{Vander\ Waals}} = (3.6)\left(\frac{\mathrm{P(atm)}}{\mathrm{T_g}^{0.7}(\mathrm{K})}\right) \tag{S4}$$

where $\mathrm{T} = 300\mathrm{K}$ and $\mathrm{P} = 1\mathrm{atm}$. In the Fig. 5 the typical Voigt-function fitting of the H_β experimental profile at the voltage of 7kV is shown. Electron density was obtained $1.994 \times 10^{15}\mathrm{cm}^{-3}$ and $2.154 \times 10^{15}\mathrm{cm}^{-3}$ at voltages 7kV for anodic and cathodic plasmas respectively.

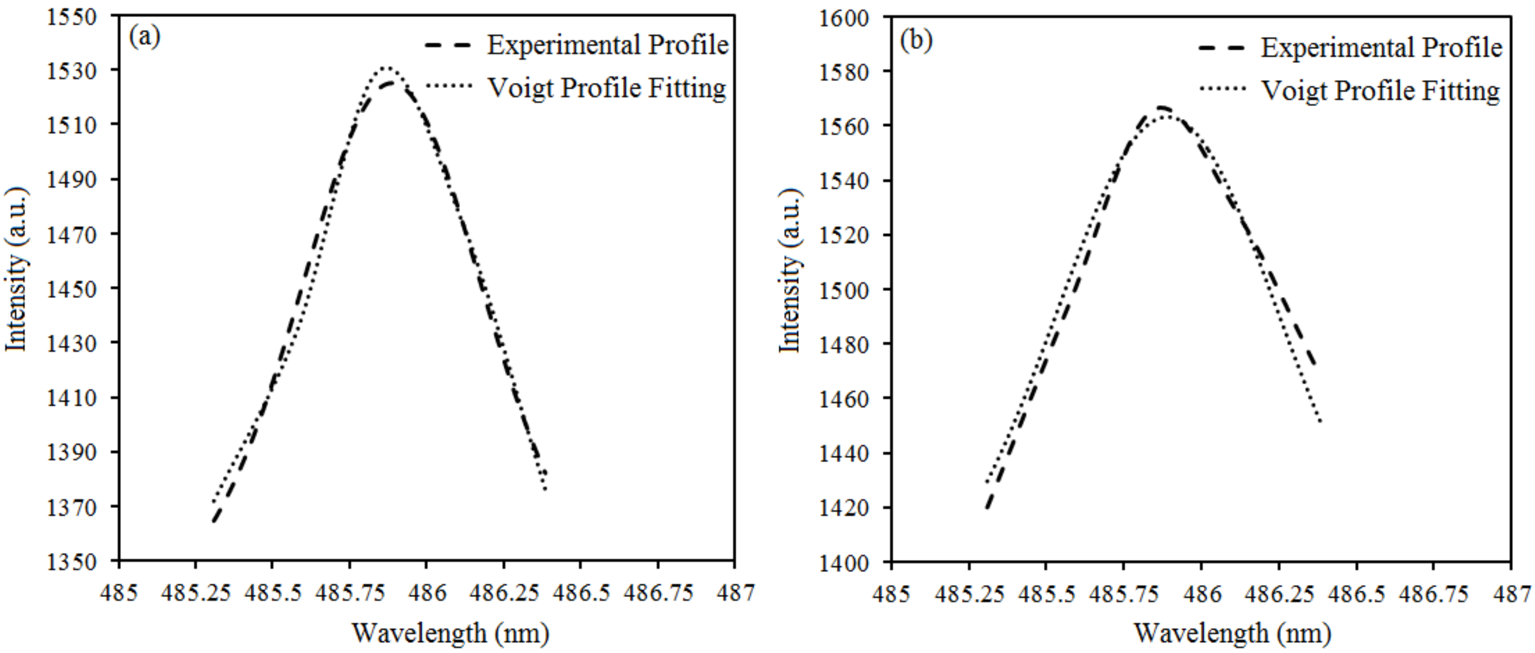

Fig S2. The typical Voigt-function fitting of the H_β experimental profile of N I spectral lines used to measure plasma density at 7kV for (a) anodic plasma and (b) cathodic plasma.

Fig. S3 shows the glass wall reactor covered in a thin layer of WO_3 powder.

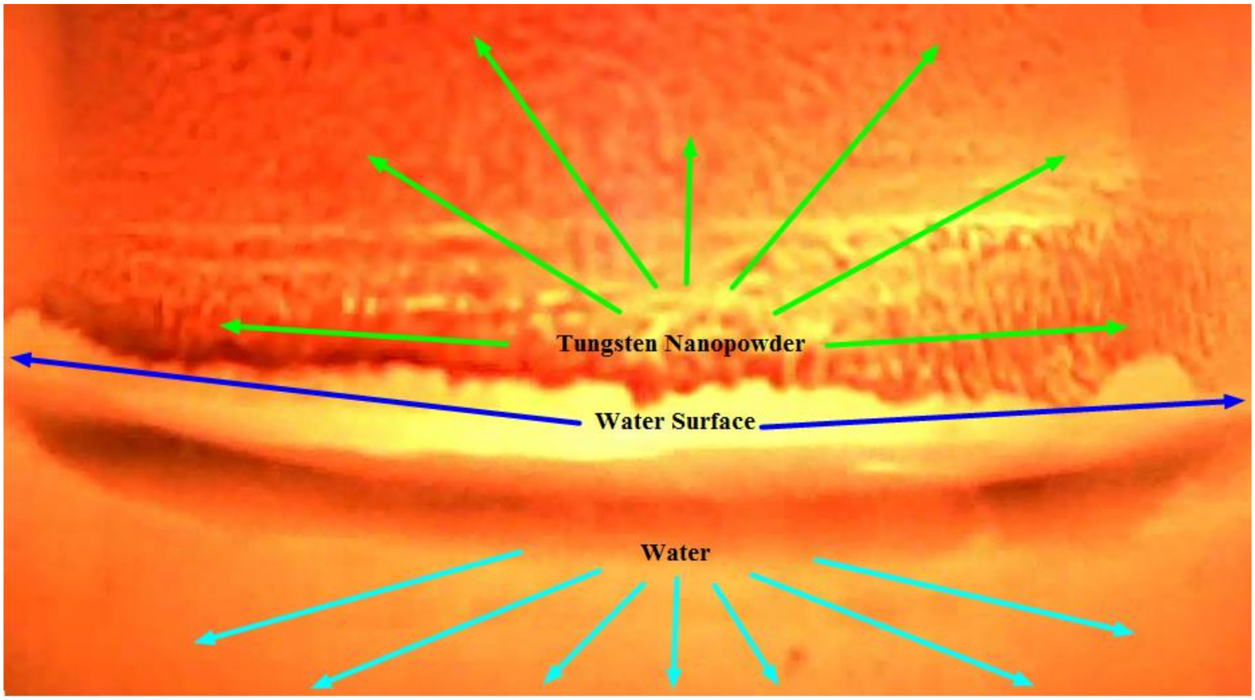

Fig S3. The thin layer of WO_3 powder on the walls of the glass reactor chamber during applying nitrogen plasma to the water surface.

Fig. S4 shows the EDS-Mapping analysis of WO_3, which confirms the uniform dispersion of tungsten and oxygen in nanoparticles.

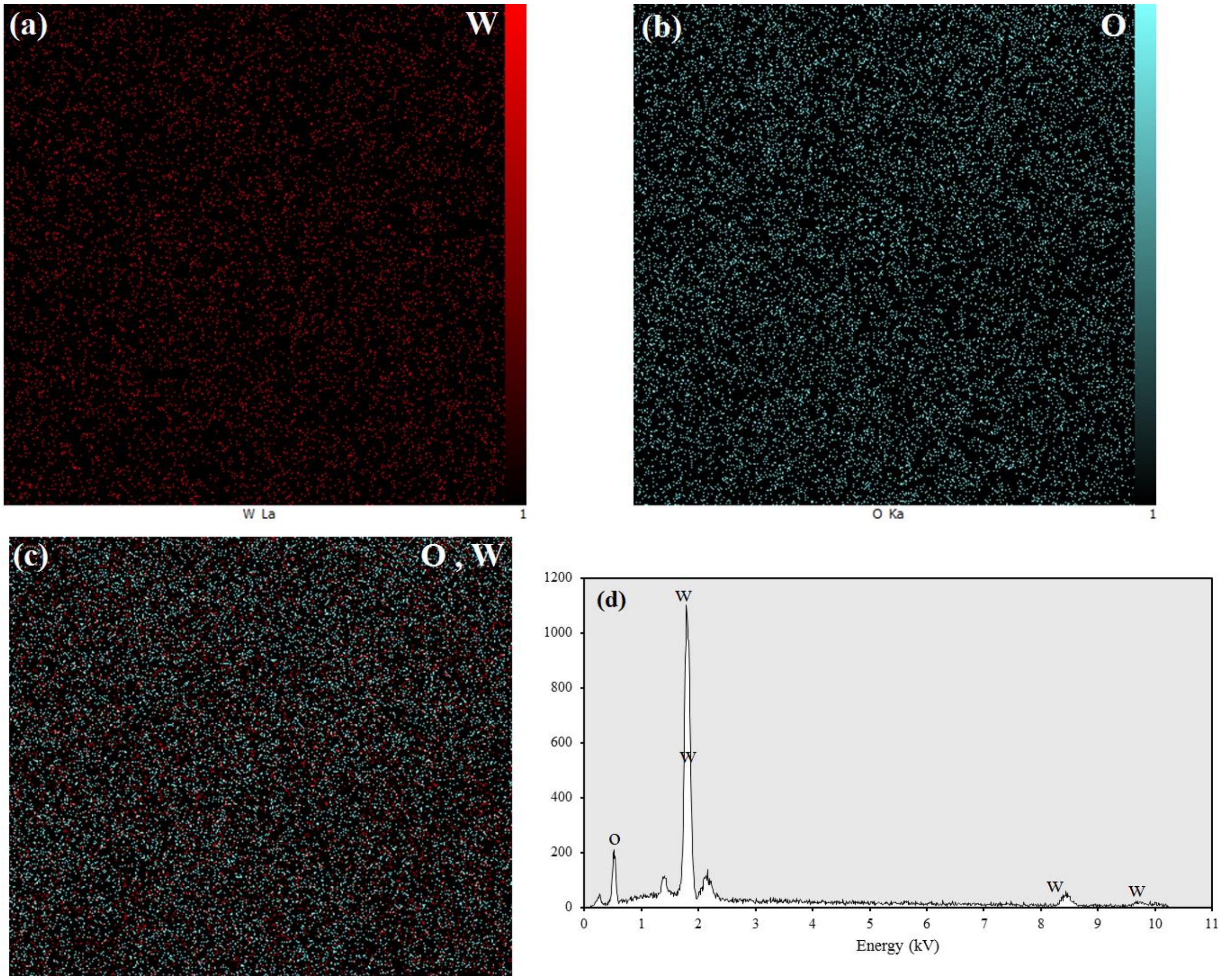

Fig S4. EDS-Mapping analysis of WO_3, (a–c) corresponding elemental mapping images and (d) the energy-dispersive X-ray (EDX) analysis of W and O elements for WO_3 nanoparticles prepared via a plasma process.

Elemental composition of obtained WO_3 nanoparticles resulting of EDS is determined in table 2.

Table. 2: Elemental composition of WO_3 nanoparticles.

**Element**	**Line**	**Weight%**	**Atomic%**
O	Ka	25.71	**79.90**
W	La	74.29	**20.10**
Total	-	100.00	**100.00**

References

[1] Baharlounezhad, F.; Mohammadi, M.A.; Zakerhamidi, M.S.; Plasma Synthesis of Ammonia by Asymmetric Electrode Arrangement, Mater. Manuf. Process. 2023, 38, 159-169, DOI: 10.1080/10426914.2022.2105875.

[2] https://physics.nist.gov/PhysRefData/ASD/lines_form.html.
